# Hexaaqua­nickel(II) 4,4′-(1,2-dihy­droxy­ethane-1,2-di­yl)dibenzoate monohydrate

**DOI:** 10.1107/S1600536810039917

**Published:** 2010-10-09

**Authors:** Shi-Jie Li, Shi-Wei Hu, Wen-Dong Song, Pei-Wen Qin, Xiao-Tian Ma

**Affiliations:** a College of Food Science and Technology, Guangdong Ocean University, Zhanjiang 524088, People’s Republic of China; b College of Science, Guangdong Ocean University, Zhanjiang 524088, People’s Republic of China; c College of Agriculture, Guang Dong Ocean University, Zhanjiang 524088, People’s Republic of China

## Abstract

In the title compound, [Ni(H_2_O)_6_](C_16_H_12_O_6_)·H_2_O, the Ni^II^ cation is located on a mirror plane and is coordinated by six water mol­ecules, two of which are also located on the mirror plane, in a distorted octa­hedral geometry. The 4,4′-(1,2-dihy­droxy­ethane-1,2-di­yl)dibenzoate anion is centrosymmetric with the mid-point of the central ethane C—C bond located on an inversion center. The uncoordinated water mol­ecule is located on a mirror plane. Extensive O—H⋯O hydrogen bonding is present in the crystal structure.

## Related literature

For metal-organic networks constructed from benzene–multicarboxyl­ate ligands, see: Wisser *et al.* (2008 ▶); Sun *et al.* (2006 ▶); Janiak (2003 ▶). For structures incorporating benzene-1,4-dicarboxyl­ate, see: Carton *et al.* (2007 ▶); Manna *et al.* (2007 ▶); Banerjee *et al.* (2005 ▶).
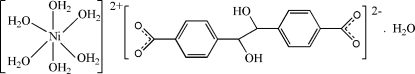


## Experimental

### 

#### Crystal data

[Ni(H_2_O)_6_](C_16_H_12_O_6_)·H_2_O
*M* *_r_* = 485.08
Monoclinic, 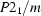

*a* = 6.0189 (12) Å
*b* = 20.436 (4) Å
*c* = 8.6096 (17) Å
β = 103.95 (3)°
*V* = 1027.8 (4) Å^3^
*Z* = 2
Mo *K*α radiation
μ = 1.01 mm^−1^
*T* = 293 K
0.30 × 0.25 × 0.21 mm

#### Data collection

Rigaku/MSC Mercury CCD diffractometer
Absorption correction: multi-scan (*REQAB*; Jacobson, 1998 ▶) *T*_min_ = 0.751, *T*_max_ = 0.816
9071 measured reflections
2120 independent reflections
2024 reflections with *I* > 2σ(*I*)
*R*_int_ = 0.036

#### Refinement

*R*[*F* ^2^ > 2σ(*F* ^2^)] = 0.071
*wR*(*F* ^2^) = 0.184
*S* = 1.03
2120 reflections
142 parameters
H-atom parameters constrained
Δρ_max_ = 0.56 e Å^−3^
Δρ_min_ = −0.54 e Å^−3^

### 

Data collection: *RAPID-AUTO* (Rigaku, 1998 ▶); cell refinement: *RAPID-AUTO*; data reduction: *CrystalStructure* (Rigaku/MSC, 2002 ▶); program(s) used to solve structure: *SHELXS97* (Sheldrick, 2008 ▶); program(s) used to refine structure: *SHELXL97* (Sheldrick, 2008 ▶); molecular graphics: *ORTEPII* (Johnson, 1976 ▶); software used to prepare material for publication: *SHELXL97*.

## Supplementary Material

Crystal structure: contains datablocks I, global. DOI: 10.1107/S1600536810039917/xu5046sup1.cif

Structure factors: contains datablocks I. DOI: 10.1107/S1600536810039917/xu5046Isup2.hkl

Additional supplementary materials:  crystallographic information; 3D view; checkCIF report

## Figures and Tables

**Table 1:** Hydrogen-bond geometry (Å, °)

*D*—H⋯*A*	*D*—H	H⋯*A*	*D*⋯*A*	*D*—H⋯*A*
O3—H10⋯O1^i^	0.89	1.94	2.810 (6)	168
O1*W*—H1*W*⋯O1^ii^	0.84	1.87	2.684 (5)	162
O2*W*—H3*W*⋯O1	0.84	2.01	2.821 (6)	163
O2*W*—H4*W*⋯O2^ii^	0.84	1.83	2.667 (6)	174
O3*W*—H5*W*⋯O5*W*^iii^	0.84	2.06	2.724 (10)	136
O3*W*—H6*W*⋯O1*W*^iv^	0.84	1.98	2.783 (8)	161
O4*W*—H7*W*⋯O5*W*^v^	0.84	2.23	3.017 (8)	157
O4*W*—H8*W*⋯O3^vi^	0.84	2.05	2.840 (6)	157
O5*W*—H9*W*⋯O2	0.84	1.95	2.776 (8)	168

Symmetry codes: (i) 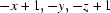; (ii) 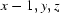; (iii) 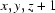; (iv) 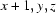; (v) 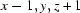; (vi) 
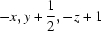
.

**Fig. 1.**
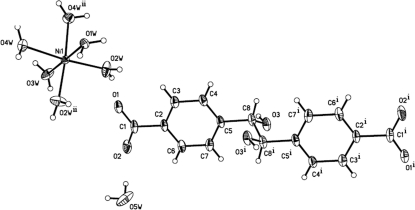
The title compound, with the atom-numbering scheme. Non-H atoms are shown with 30% probability displacement ellipsoids (H atoms are represented by arbitrary spheres). [Symmetry codes: (i) -x, -y, -z; (ii) x, 0.5 - y, z.]

**Fig. 2.**
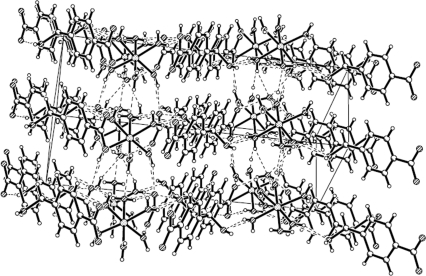
The packing and hydrogen bonding of the title compound.

## supplementary crystallographic information

### Comment

Metal-organic networks constructed by benzene-multicarboxylato ligands, have attracted a great deal of recent interest (Wisser *et al.*, 2008; Sun *et al.*, 2006; Janiak *et al.*, 2003). Benzene-1,4-dicarboxylate with a 180° angle between the two carboxylic groups, can form short bridges *via* one carboxylato end and long bridges *via* the benzene ring, leading to a great variety of novel structures (Carton *et al.*, 2007; Manna *et al.*, 2007; Banerjee *et al.*, 2005). Considering that in mind, our group select a derivative of the benzene-1,4-dicarboxylate named 4,4'-(1,2-dihydroxyethane-1,2-diyl)dibenzoate to react with Ni(NO3)_2_ to obtain novel metal-organic complex.

In figure 1, the title compound (C_16_H_12_O_6_)[Ni6H_2_O].H_2_O is obtained under hydrothermal condition, which comprises one 4,4'-(1,2-dihydroxyethane-1,2 -diyl)dibenzoate anion, one [Ni6H_2_O]^2+^ cation and a solvent water molecule, of which the [Ni6H_2_O]^2+^ cation and solvent water is lying on mirror planes, and the anion is locating on an inversion center. the two carboxyl groups of the ligand are total deprotonated, indicated by a difference of the bond lengths, which are also lying in the plane of the benzene rings. and the Ni^II^ center is coordinated by six water molecules instead of the 4,4'-(1,2-dihydroxyethane- 1,2-diyl)dibenzoate ligand. the O—H···O hydrogen bonding interactions between the carboxyl and hydroxyl of the ligands build an infinite chain along *a* axis. the chains, [Ni6H_2_O]^2+^ cations and solvent water molecules was further linked by additional O—H···O hydrogen bonds, forming a three-dimensional network.

### Experimental

A solution of 4,4'-(1,2-dihydroxyethane-1,2-diyl)dibenzoic acid (0.5 mol, 0.15 g) and Ni(NO_3_)_2_ (0.5 mol, 0.14 g) and 20 ml water was stirred continuously, whose pH was adjusted to 7 by the addition of NaOH solution. The solution was then sealed in an autoclave equipped with a Teflon liner (20 ml) and heated at 373 K for 4 days. Crystals of the title compound were obtained by slow evaporation at room temperature.

### Refinement

H atoms bound to C atoms were placed at calculated positions and were treated as riding on the parent atoms with C—H = 0.93 Å (aromatic) and 0.98 Å (CH) and with *U*_iso_(H) = 1.2 *U*_eq_(C). H atoms of hydroxyl group and water molecules were located in a difference Fourier map and refined as riding with O—H = 0.85+_0.01 Å and *U*_iso_(H) = 1.5*U*_eq_(O) for water O atoms and O—H = 0.89±0.01 Å and 1.2 *U*_eq_(O) for hydroxyl.

### Crystal data

**Table:**

[Ni(H_2_O)_6_](C_16_H_12_O_6_)·H_2_O	*F*(000) = 508
*M**_r_* = 485.08	*D*_x_ = 1.567 Mg m^−^^3^
Monoclinic, *P*2_1_/*m*	Mo *K*α radiation, λ = 0.71073 Å
Hall symbol: -P 2yb	Cell parameters from 3600 reflections
*a* = 6.0189 (12) Å	θ = 1.4–28°
*b* = 20.436 (4) Å	µ = 1.01 mm^−^^1^
*c* = 8.6096 (17) Å	*T* = 293 K
β = 103.95 (3)°	Block, green
*V* = 1027.8 (4) Å^3^	0.30 × 0.25 × 0.21 mm
*Z* = 2	

### Data collection

**Table:**

Rigaku/MSC Mercury CCD diffractometer	2120 independent reflections
Radiation source: fine-focus sealed tube	2024 reflections with *I* > 2σ(*I*)
graphite	*R*_int_ = 0.036
ω scans	θ_max_ = 26.2°, θ_min_ = 3.2°
Absorption correction: multi-scan (REQAB; Jacobson, 1998)	*h* = −7→7
*T*_min_ = 0.751, *T*_max_ = 0.816	*k* = −25→25
9071 measured reflections	*l* = −10→9

### Refinement

**Table:**

Refinement on *F*^2^	Primary atom site location: structure-invariant direct methods
Least-squares matrix: full	Secondary atom site location: difference Fourier map
*R*[*F*^2^ > 2σ(*F*^2^)] = 0.071	Hydrogen site location: inferred from neighbouring sites
*wR*(*F*^2^) = 0.184	H-atom parameters constrained
*S* = 1.03	*w* = 1/[σ^2^(*F*_o_^2^) + (0.040*P*)^2^ + 10.*P*] where *P* = (*F*_o_^2^ + 2*F*_c_^2^)/3
2120 reflections	(Δ/σ)_max_ < 0.001
142 parameters	Δρ_max_ = 0.56 e Å^−^^3^
0 restraints	Δρ_min_ = −0.54 e Å^−^^3^

### Special details

**Table:**

Geometry. All e.s.d.'s (except the e.s.d. in the dihedral angle between two l.s. planes) are estimated using the full covariance matrix. The cell e.s.d.'s are taken into account individually in the estimation of e.s.d.'s in distances, angles and torsion angles; correlations between e.s.d.'s in cell parameters are only used when they are defined by crystal symmetry. An approximate (isotropic) treatment of cell e.s.d.'s is used for estimating e.s.d.'s involving l.s. planes.
Refinement. Refinement of *F*^2^ against ALL reflections. The weighted *R*-factor *wR* and goodness of fit *S* are based on *F*^2^, conventional *R*-factors *R* are based on *F*, with *F* set to zero for negative *F*^2^. The threshold expression of *F*^2^ > σ(*F*^2^) is used only for calculating *R*-factors(gt) *etc*. and is not relevant to the choice of reflections for refinement. *R*-factors based on *F*^2^ are statistically about twice as large as those based on *F*, and *R*- factors based on ALL data will be even larger.

### Fractional atomic coordinates and isotropic or equivalent isotropic displacement parameters (Å^2^)

**Table:**

	*x*	*y*	*z*	*U*_iso_*/*U*_eq_	
Ni1	0.14235 (16)	0.2500	0.95708 (11)	0.0242 (3)	
O1W	−0.2105 (8)	0.2500	0.8578 (6)	0.0279 (11)	
H1W	−0.2488	0.2152	0.8065	0.042*	
O2W	0.1795 (7)	0.1762 (3)	0.8058 (6)	0.0555 (14)	
H3W	0.2947	0.1584	0.7850	0.083*	
H4W	0.0631	0.1713	0.7301	0.083*	
O3W	0.4872 (10)	0.2500	1.0567 (7)	0.0504 (18)	
H5W	0.5246	0.2500	1.1572	0.076*	
H6W	0.6021	0.2500	1.0175	0.076*	
O4W	0.0917 (8)	0.3180 (2)	1.1214 (5)	0.0439 (11)	
H7W	0.0201	0.3101	1.1918	0.066*	
H8W	0.0439	0.3509	1.0647	0.066*	
O1	0.5811 (7)	0.1431 (2)	0.7095 (5)	0.0438 (11)	
O2	0.8274 (7)	0.1532 (3)	0.5570 (6)	0.0559 (14)	
O3	0.1710 (8)	−0.0731 (2)	0.0238 (6)	0.0478 (12)	
H10	0.2666	−0.0921	0.1056	0.072*	
C1	0.6426 (10)	0.1337 (3)	0.5802 (8)	0.0404 (15)	
C2	0.4864 (9)	0.0953 (3)	0.4462 (7)	0.0352 (13)	
C3	0.2822 (10)	0.0692 (3)	0.4681 (8)	0.0401 (14)	
H1	0.2394	0.0765	0.5635	0.048*	
C4	0.1421 (10)	0.0323 (3)	0.3473 (7)	0.0397 (14)	
H2	0.0051	0.0155	0.3621	0.048*	
C5	0.2046 (10)	0.0205 (3)	0.2063 (7)	0.0364 (14)	
C6	0.5483 (10)	0.0834 (3)	0.3050 (8)	0.0416 (15)	
H4	0.6852	0.1004	0.2903	0.050*	
C7	0.4090 (11)	0.0465 (3)	0.1839 (8)	0.0432 (15)	
H3	0.4520	0.0392	0.0886	0.052*	
C8	0.0514 (10)	−0.0204 (3)	0.0746 (7)	0.0389 (14)	
H9	−0.0740	−0.0384	0.1158	0.047*	
O5W	0.8270 (14)	0.2500	0.3307 (9)	0.097 (4)	
H9W	0.8455	0.2192	0.3971	0.145*	

### Atomic displacement parameters (Å^2^)

**Table:**

	*U*^11^	*U*^22^	*U*^33^	*U*^12^	*U*^13^	*U*^23^
Ni1	0.0219 (5)	0.0290 (5)	0.0213 (5)	0.000	0.0045 (3)	0.000
O1W	0.024 (2)	0.026 (3)	0.032 (3)	0.000	0.003 (2)	0.000
O2W	0.028 (2)	0.076 (4)	0.056 (3)	0.008 (2)	−0.003 (2)	−0.036 (3)
O3W	0.027 (3)	0.099 (6)	0.021 (3)	0.000	−0.003 (2)	0.000
O4W	0.048 (2)	0.045 (3)	0.037 (2)	−0.002 (2)	0.0068 (19)	−0.010 (2)
O1	0.036 (2)	0.041 (2)	0.045 (3)	−0.0026 (19)	−0.0069 (19)	−0.011 (2)
O2	0.032 (2)	0.071 (3)	0.058 (3)	−0.013 (2)	−0.002 (2)	−0.027 (3)
O3	0.050 (3)	0.030 (2)	0.052 (3)	0.004 (2)	−0.010 (2)	−0.007 (2)
C1	0.033 (3)	0.033 (3)	0.045 (4)	0.005 (3)	−0.012 (3)	−0.014 (3)
C2	0.029 (3)	0.028 (3)	0.039 (3)	0.003 (2)	−0.010 (2)	−0.009 (2)
C3	0.038 (3)	0.036 (3)	0.040 (3)	−0.001 (3)	−0.003 (3)	−0.006 (3)
C4	0.036 (3)	0.035 (3)	0.041 (3)	−0.007 (3)	−0.006 (3)	−0.003 (3)
C5	0.035 (3)	0.022 (3)	0.041 (3)	0.001 (2)	−0.013 (2)	−0.002 (2)
C6	0.031 (3)	0.039 (3)	0.047 (4)	−0.001 (3)	−0.005 (3)	−0.013 (3)
C7	0.039 (3)	0.041 (3)	0.041 (3)	0.002 (3)	−0.007 (3)	−0.010 (3)
C8	0.036 (3)	0.028 (3)	0.044 (3)	0.000 (2)	−0.009 (3)	−0.008 (3)
O5W	0.062 (5)	0.197 (12)	0.033 (4)	0.000	0.015 (4)	0.000

### Geometric parameters (Å, °)

**Table:**

Ni1—O2W^i^	2.039 (5)	O3—H10	0.8851
Ni1—O2W	2.039 (4)	C1—C2	1.519 (7)
Ni1—O3W	2.046 (6)	C2—C6	1.376 (9)
Ni1—O4W	2.058 (4)	C2—C3	1.393 (9)
Ni1—O4W^i^	2.058 (4)	C3—C4	1.392 (8)
Ni1—O1W	2.090 (5)	C3—H1	0.9300
O1W—H1W	0.8400	C4—C5	1.377 (9)
O2W—H3W	0.8400	C4—H2	0.9300
O2W—H4W	0.8400	C5—C7	1.395 (9)
O3W—H5W	0.8400	C5—C8	1.526 (7)
O3W—H6W	0.8400	C6—C7	1.393 (8)
O4W—H7W	0.8400	C6—H4	0.9300
O4W—H8W	0.8398	C7—H3	0.9300
O1—C1	1.269 (8)	C8—C8^ii^	1.531 (12)
O2—C1	1.242 (8)	C8—H9	0.9800
O3—C8	1.422 (7)	O5W—H9W	0.8396
			
O2W^i^—Ni1—O2W	95.4 (3)	O2—C1—C2	117.2 (6)
O2W^i^—Ni1—O3W	90.66 (17)	O1—C1—C2	119.1 (6)
O2W—Ni1—O3W	90.66 (17)	C6—C2—C3	119.2 (5)
O2W^i^—Ni1—O4W	89.8 (2)	C6—C2—C1	120.8 (6)
O2W—Ni1—O4W	174.6 (2)	C3—C2—C1	120.0 (6)
O3W—Ni1—O4W	90.87 (18)	C4—C3—C2	120.1 (6)
O2W^i^—Ni1—O4W^i^	174.6 (2)	C4—C3—H1	119.9
O2W—Ni1—O4W^i^	89.8 (2)	C2—C3—H1	119.9
O3W—Ni1—O4W^i^	90.87 (18)	C5—C4—C3	120.5 (6)
O4W—Ni1—O4W^i^	85.0 (3)	C5—C4—H2	119.7
O2W^i^—Ni1—O1W	89.75 (16)	C3—C4—H2	119.7
O2W—Ni1—O1W	89.75 (16)	C4—C5—C7	119.5 (5)
O3W—Ni1—O1W	179.4 (2)	C4—C5—C8	120.4 (6)
O4W—Ni1—O1W	88.68 (17)	C7—C5—C8	120.1 (6)
O4W^i^—Ni1—O1W	88.68 (17)	C2—C6—C7	121.0 (6)
Ni1—O1W—H1W	109.7	C2—C6—H4	119.5
Ni1—O2W—H3W	132.8	C7—C6—H4	119.5
Ni1—O2W—H4W	112.6	C6—C7—C5	119.7 (6)
H3W—O2W—H4W	111.1	C6—C7—H3	120.2
Ni1—O3W—H5W	115.1	C5—C7—H3	120.2
Ni1—O3W—H6W	133.0	O3—C8—C5	112.6 (5)
H5W—O3W—H6W	111.9	O3—C8—C8^ii^	106.6 (7)
Ni1—O4W—H7W	123.6	C5—C8—C8^ii^	112.0 (6)
Ni1—O4W—H8W	103.2	O3—C8—H9	108.5
H7W—O4W—H8W	114.1	C5—C8—H9	108.5
C8—O3—H10	111.6	C8^ii^—C8—H9	108.5
O2—C1—O1	123.7 (5)		
			
O2—C1—C2—C6	−0.5 (9)	C3—C2—C6—C7	0.7 (9)
O1—C1—C2—C6	−179.2 (6)	C1—C2—C6—C7	177.9 (6)
O2—C1—C2—C3	176.7 (6)	C2—C6—C7—C5	−0.6 (10)
O1—C1—C2—C3	−2.0 (9)	C4—C5—C7—C6	0.6 (9)
C6—C2—C3—C4	−0.7 (9)	C8—C5—C7—C6	−179.6 (5)
C1—C2—C3—C4	−178.0 (6)	C4—C5—C8—O3	−126.7 (6)
C2—C3—C4—C5	0.8 (9)	C7—C5—C8—O3	53.5 (8)
C3—C4—C5—C7	−0.7 (9)	C4—C5—C8—C8^ii^	113.2 (8)
C3—C4—C5—C8	179.6 (5)	C7—C5—C8—C8^ii^	−66.6 (9)

Symmetry codes: (i) *x*, −*y*+1/2, *z*; (ii) −*x*, −*y*, −*z*.

### Hydrogen-bond geometry (Å, °)

**Table:**

*D*—H···*A*	*D*—H	H···*A*	*D*···*A*	*D*—H···*A*
O3—H10···O1^iii^	0.89	1.94	2.810 (6)	168
O1W—H1W···O1^iv^	0.84	1.87	2.684 (5)	162
O2W—H3W···O1	0.84	2.01	2.821 (6)	163
O2W—H4W···O2^iv^	0.84	1.83	2.667 (6)	174
O3W—H5W···O5W^v^	0.84	2.06	2.724 (10)	136
O3W—H6W···O1W^vi^	0.84	1.98	2.783 (8)	161
O4W—H7W···O5W^vii^	0.84	2.23	3.017 (8)	157
O4W—H8W···O3^viii^	0.84	2.05	2.840 (6)	157
O5W—H9W···O2	0.84	1.95	2.776 (8)	168

Symmetry codes: (iii) −*x*+1, −*y*, −*z*+1; (iv) *x*−1, *y*, *z*; (v) *x*, *y*, *z*+1; (vi) *x*+1, *y*, *z*; (vii) *x*−1, *y*, *z*+1; (viii) −*x*, *y*+1/2, −*z*+1.
